# Clinical characteristics and outcomes of obstetric patients requiring ICU admission

**DOI:** 10.1186/cc9933

**Published:** 2011-03-11

**Authors:** H El-Abd, K Mashhour, A Mwafy

**Affiliations:** 1 Cairo University Hospitals, Cairo, Egypt

## Introduction

Despite therapeutic advances during this century, maternal mortality remains an important public health problem. So it was logical to study these patients who were referred from the Gynecology and Obstetric Department to our ICU aiming to review a series of these patients in order to assess the spectrum of diseases, required interventions, complications that occurred and maternal mortality and to identify conditions associated with maternal death.

## Methods

A retrospective cohort study in the Critical Care Medicine Department, Cairo University. The medical records of all obstetric ICU admissions over the period from January 2005 to December 2009 were reviewed.

## Results

Over these 5 years, 169 women required ICU admission (1.6% of all ICU admissions). The mean age was 29.29 ± 6.06 years; mean gestational age was 34.56 ± 3.01 weeks, and the mean length of ICU stay was 3.32 ± 3.6 days. Most patients (77%) were admitted with obstetric cause, the most common cause of maternal morbidity was pregnancy-induced hypertension (56.21%), followed by obstetric hemorrhage (17.75%). Heart failure (13.6%) was the principal nonobstetric cause. Maternal mortality rate was 4.14%, with hypovolemic shock and MODS (71.4%) as main causes. Despite the incidence of death being higher among patients with obstetric versus nonobstetric cause (4.6% and 2.6%, respectively), this was not statistically significant (*P *= 0.91). Twenty-five percent of patients had prior medical diseases, 76.74% of them had cardiac problems. The most common interventions were central venous catheterization (91.1%), endotracheal tube intubation (16.6%), and mechanical ventilation (12.4%). Disturbed conscious level, MODS, shock, ARF, bleeding, and ARDS were present in 17.8%, 12.4%, 10.7%, 10.7%, 8.9% and 7.1% of patients, respectively. Anemia, leucocytosis, and thrombocytopenia were more present in the obstetric group.

## Conclusions

The admission rate to the ICU may be reduced by improving the management of the hypertensive disease during pregnancy. Early admission to the ICU decreases the maternal mortality and morbidity. Despite several complications occurring with obstetric patients, the prognosis is still good.
